# The complete chloroplast genome of *Solanum berthaultii*, one of the potato wild relative species

**DOI:** 10.1080/23802359.2017.1285213

**Published:** 2017-02-06

**Authors:** Tae-Ho Park

**Affiliations:** a Department of Horticulture, Daegu University, Gyeongsan, South Korea;; b Institute of Life and Environment, Daegu University, Gyeongsan, South Korea

**Keywords:** Chloroplast, genome, genome sequence, *Solanum berthaultii*

## Abstract

*Solanum berthaultii* is a wild species belonging to Solanaceae family. The complete chloroplast genome of *S. berthaultii* was constituted by *de novo* assembly using a small amount of whole genome sequencing data. The chloroplast genome of *S. berthaultii* was 155,533 bp in length and consisted of 25,593 bp of a pair of inverted repeats, 18,372 bp of small single copy and 85,975 bp of large single-copy regions. 158 genes were annotated including 105 protein-coding, 45 tRNA, and 8 rRNA genes. Maximum-likelihood phylogenetic analysis with eight Solanaceae species revealed that *S. berthaultii* is most closely grouped with *S. tuberosum*.

*Solanum berthaultii*, a wild diploid species originating from Bolivia, South America is a relative to potato (*S. tuberosum*). It was identified as a source of resistance to several important pathogens such as *Phytophthora infestans*, Potato virus Y etc. and used for potato breeding (Ewing et al. 2000; Park et al. 2009; Rauscher et al. 2010; Tan et al. 2010; Nouri-Ellouz et al. 2016). However, interspecific sexual hybridization is limited due to its sexual incompatibility with *S. tuberosum* caused by the different ploidy levels of the genome and endosperm balance number (Ortiz & Ehlenfeldt 1992; Cho et al. 1997). Indeed, crop improvement via protoplast fusion with the two different species has been attempted to overcome sexual barriers for interspecific gene transfer (Bidani et al. 2007; Nouri-Ellouz et al. 2016) and it is necessary to confirm whether or not plastome fusion occurs with sequence information of chloroplast in potato breeding program (Cho & Park 2016; Cho et al. 2016).

The *S. berthaultii* (PI310981) was provided by Highland Agriculture Research Institute, South Korea. An Illumina paired-end (PE) genomic library was constructed with total genomic DNA according to the PE standard protocol (Illumina, San Diego, CA) and sequenced using an Illumina HiSeq2000 at Macrogen (http://www.macrogen.com/kor/). Low-quality bases with raw scores of 20 or less were removed and then approximately 2.5 Gbp of high-quality of PE reads were assembled by a CLC genome assembler (CLC Inc, Rarhus, Denmark) (Kim et al. 2015). The principal contigs representing the chloroplast genome were retrieved from the total contigs using Nucmer (Kurtz et al. 2004) with the chloroplast genome sequence of *S. tuberosum* (KM489056) as the reference sequence (Cho et al. 2016). The representative chloroplast contigs were arranged in order based on BLASTZ analysis (Schwartz et al. 2003) with the reference sequence and connected to a single draft sequence by joining the overlapping terminal sequences. The chloroplast genes were predicted using DOGMA (Wyman et al. 2004) and BLAST searches.

The complete chloroplast genome of *S. berthaultii* (GenBank accession no. KY419708) was 155,533 bp in length including 25,593 bp inverted repeats (IRa and IRb) regions separated by small single copy (SSC) region of 18,372 bp and large single copy (LSC) region of 85,975 bp with the typical quadripartite structure of most plastids, and the structure and gene features were typically identical to those of higher plants. A total of 158 genes with an average size of 583 bp were annotated including 105 protein-coding genes with an average size of 765 bp, 45 tRNA genes, and 8 rRNA genes. An overall GC content was 37.88%.

Phylogenetic analysis was performed using chloroplast coding sequences of *S. berthaultii* and those of seven species including *S. lycopersicum* (Daniell et al. 2006) and *Capsicum annuum* (Jo et al. 2011) in Solanaceae family by a maximum-likelihood method in MEGA 6.0 (Tamura et al. 2013). According to the phylogenetic tree, *S. berthaultii* belonged to the same clade in *Solanum* species as expected and interestingly it was most closely grouped with *S. tuberosum* (Figure 1).

**Figure 1.**
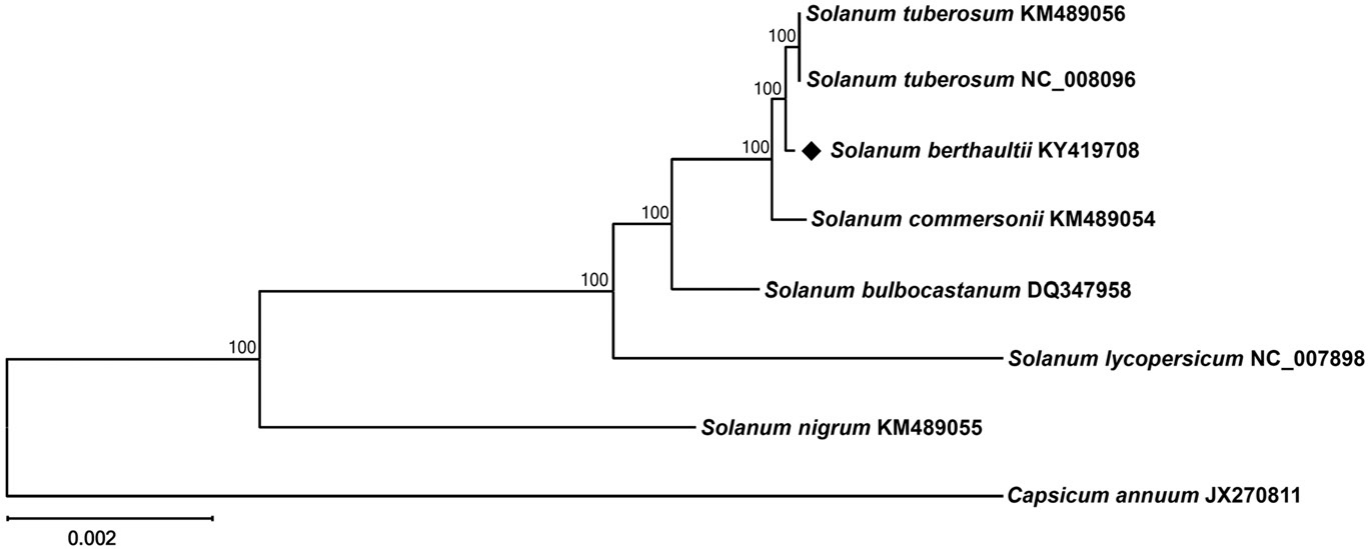
Maximum-likelihood phylogenetic tree of *S. berthaultii* with eight species belonging to the Solanaceae based on chloroplast protein-coding sequences. Numbers in the nodes are the bootstrap values from 1000 replicates.
